# Tridimensional model of CBT: a transdiagnostic and transtheoretical pragmatic model

**DOI:** 10.3389/fpsyg.2025.1555047

**Published:** 2025-05-30

**Authors:** Kadir Özdel, Mehmet Hakan Turkcapar

**Affiliations:** ^1^University of Health Sciences, Ankara Etlik City Hospital, Psychiatry Clinic, Istanbul, Türkiye; ^2^Department of Psychology, Social Sciences University of Ankara, Ankara, Türkiye

**Keywords:** cognitive behavioral therapy, attention, schema, emotions, integration

## Abstract

Cognitive Behavioral Therapies (CBT) essentially represent an umbrella concept. Behavioral and cognitive (schema) theories form the foundation of all CBT approaches. However, the explanations and methods proposed by the models under the CBT umbrella sometimes contradict one another. We believe that a practical and pragmatic model integrating these CBT models will contribute significantly to the field. Upon reviewing the literature, we conclude that three individual strategies across three domains play a key role in the persistence of all psychopathologies: the attention/focus domain, the cognitive evaluation/operation domain, and the behavioral domain. Although individuals experiencing emotional difficulties utilize efforts in these three interrelated domains to solve their problems, these efforts often exacerbate the issues. The Tridimensional CBT (TriD-CBT) model recommends identifying the controllable components within these three domains and focusing interventions on them. In this article, we aim to present the general theoretical framework of the TriD-CBT model in light of the existing literature.

## Introduction

The term “Cognitive Behavioral Therapies” (CBT) serves as an umbrella term encompassing numerous models that place learning at the center of the emergence, maintenance, and recovery of mental disorders (McMain et al., 2015). Two cornerstone schools of thought—behaviorism and cognitive theory (schema theory)—form the theoretical foundation of CBT. All models under the CBT umbrella are, in some way, based on these two theoretical backgrounds. Clinically observed phenomena and the methods used in treatment are explained and named according to these schools of thought (Ertmer and Newby, 2013).

The effectiveness of CBT for a wide range of psychological conditions, across diverse populations and contexts, has been extensively researched and is well supported by scientific evidence. A meta-review suggests that CBT may even be beneficial for psychological conditions where current treatment evidence is limited (Fordham et al., 2021). As the most scientifically studied form of psychotherapy (Hofmann et al., 2012), CBT is also the approach in which theoretical models and mechanisms of change have been most thoroughly examined. Furthermore, CBT aligns well with both mainstream and contemporary paradigms in human psychology (David et al., 2018). Consequently, the contemporary, evidence-based, and integrative nature of CBT enables it to be used reliably, effectively, and pragmatically within a transtheoretical model. The importance of transtheoretical psychotherapies being evidence-based, scientifically supported, and grounded in a solid theoretical foundation has been emphasized in numerous approaches and frameworks (Prochaska and DiClemente, 1982; Cole, 2022; Lutz and Rief, 2024; Lutz et al., 2024).

Although CBT is the gold standard for managing many clinical conditions, data on full recovery suggest we still have a long way to go. The overall treatment response rate for CBT ranges from 38 to 82% (Hofmann et al., 2012). When we look at relapse rates after CBT, we see similar unmet needs (Levy et al., 2021). Can we improve this situation by introducing new psychotherapies or by developing existing CBT in a holistic way? When you read the title of this article, you might think of a brand-new acronym that many practitioners may find unnecessary. The good news is that this is not a “new” model promoting itself as an alternative approach to unmet needs in psychological disorders. Instead, it is a pragmatic and integrative model within the framework of CBT. This model aims to provide practitioners with a structured framework for the evaluation, psychoeducation, intervention, and relapse prevention phases of CBT.

Norcross and Grencavage (1989) argued that the existence of hundreds of rival psychotherapy models—many of which are considered variations of CBT—does not necessarily improve therapy outcomes but instead creates a “confusing cacophony” (Norcross and Grencavage, 1989). While this claim may seem extreme, we propose that irreconcilable models do not contribute to a “symphony” in the mental health field. Theoretical eclecticism, as Lazarus and Beutler (1993) suggested, is not the antidote to the rigid orthodoxy of individual schools of thought (Lazarus and Beutler, 1993). Instead, Lazarus advocates for technical eclecticism, which involves adopting techniques from various schools and integrating them into a robust theoretical framework (Lazarus and Messer, 1991). A pragmatic framework that allows for the integration of different CBT models could help practitioners create a more “symphonic” and cohesive practice. Although there have been ongoing efforts in the field to integrate various schools of psychotherapy (Norcross and Goldfried, 2019), a more comprehensive discussion of integration is beyond the scope of this article. Some authors have even suggested that unification will ultimately be the final goal of these efforts (Magnavita et al., 2024). Much of the debate centers on the unique versus common factors of different psychotherapies. However, it appears that a dichotomous approach is neither helpful nor appropriate (McAleavey and Castonguay, 2014). While some attempts have been made to identify shared mechanisms—such as emotion regulation (Palmieri et al., 2022) or dialectical frameworks (Herzovich and Govrin, 2023) by psychodynamic and cognitive behavioral approaches—we believe that broadening the discussion too much may detract from our goal of offering a pragmatic yet grounded approach. Even determining which CBT models should be included is a complex issue. Therefore, we suggest that it is more practical to categorize them under broader headings, such as generational definitions.

To understand how various CBT models can be integrated, it is essential to review the prominent theoretical constructs emphasized throughout the historical development of CBT. Organisms’ responses are triggered by internal or external stimuli (Diamond and Aspinwall, 2003). From a behaviorist perspective, conditioning (both classical and operant) plays a central role in the emergence of these responses. In contrast, schema theory posits that schema activation is the primary driver of these reactions. Treatment, therefore, involves reconditioning or eliminating existing conditioning and reducing reinforcement for undesired behaviors from a behavioral perspective. From a cognitive perspective, the same phenomenon is explained through the activation or reconstruction of positive schemas (Padesky, 1994). In both perspectives, responses can be divided into automatic and voluntary phases. For instance, physiological reactions and behavioral tendencies are automatic, while overt and covert behaviors are voluntary. Similarly, negative automatic thoughts, intrusive thoughts, and images are automatic, whereas balanced thinking or ruminative thinking styles are, at least partially, voluntary (Horowitz, 1975). This can be based on dual processing model. Dual-process models propose two fundamental styles of information processing: automatic (reflexive) and controlled (reflective). The automatic style relies on well-learned information and heuristic cues, characterized as fast, peripheral, experiential, impulsive, and associative. In contrast, the controlled style is based on rules and symbolic logic, described as slow, deliberate, systematic, central, rational, and reflective (Claypool et al., 2012). Thus, automatic (reflexive) processing represents an involuntary component, while controlled processing reflects a voluntary component.

Although, it is considered controversial by some authors (Hofmann and Asmundson, 2008; Carona, 2023) the development of CBT is often described in terms of three generations. The first generation emphasizes behavioral reactions, the second generation focuses on cognitive processes, and the third generation highlights attentional focus (Hayes and Hofmann, 2021). Although third-generation models introduced “values” as an original contribution, these were previously addressed in humanistic therapies and can be considered another form of belief content guiding behavior. In fact, the emphasis on values as a central component of psychological well-being has long been a hallmark of humanistic approaches, which prioritize personal meaning, self-actualization, and the alignment of behavior with deeply held principles (Rogers, 1995). This perspective suggests that values are not only motivational constructs but also serve as a framework for interpreting experiences and making decisions, a notion that has been integrated into third-wave therapies to enhance their applicability and depth. Additionally, various mindfulness practices within third-wave therapies offer alternative ways of relating to internal (e.g., perceptions, feelings, thoughts, beliefs) and external stimuli (e.g., environmental events) through attentional operations (Segal et al., 2012). These practices aim to achieve a mental state in which individuals are less engaged with their inner experiences. Techniques such as attention training, worry/rumination postponement, and detached mindfulness experiments—seen in models like the metacognitive model (Wells, 2011) and acceptance and commitment therapy (Hayes and Hofmann, 2021) are examples of such operations.

### Tridimensions in emotional disorders

In light of the evaluations above, three main areas emerge as critical in the onset and continuation of both clinical and non-clinical conditions related to emotional distress: cognitive reactions and operations, behavioral responses and operations, and focus/attention. A model centered on these three areas could serve as a diagnostic and transdiagnostic framework. Rather than introducing a novel claim, the Tridimensional (TriD-CBT) model aims to integrate evidence-based models under the CBT umbrella and their associated practices into a coherent, easy-to-understand framework.

TriD-CBT, suggests that incorporating questions and integrated interventions related to these three domains—during structured assessment, in problem-level formulations, in modeling the maintenance of the problem, and throughout the intervention process—will lead to the development of more effective and user-friendly therapeutic strategies. This approach could also enhance psychoeducation for clients. While cognitive-behavioral protocols offer a wide range of techniques (Butler et al., 2006) therapist competency often becomes a concern (Creed et al., 2016). A common issue is the failure to address critical aspects of a problem when implementing techniques. For instance, a client may engage in walking as a behavioral activation strategy, but this activity may prove ineffective if the client continues to ruminate or focus inwardly. Similarly, exposure to a doorknob as part of a behavioral strategy to reduce compulsive handwashing may fail if cognitive and attentional domains are not addressed—such as focusing on external stimuli and refraining from trying to convince oneself that the doorknob is safe.

**Figure 1:**
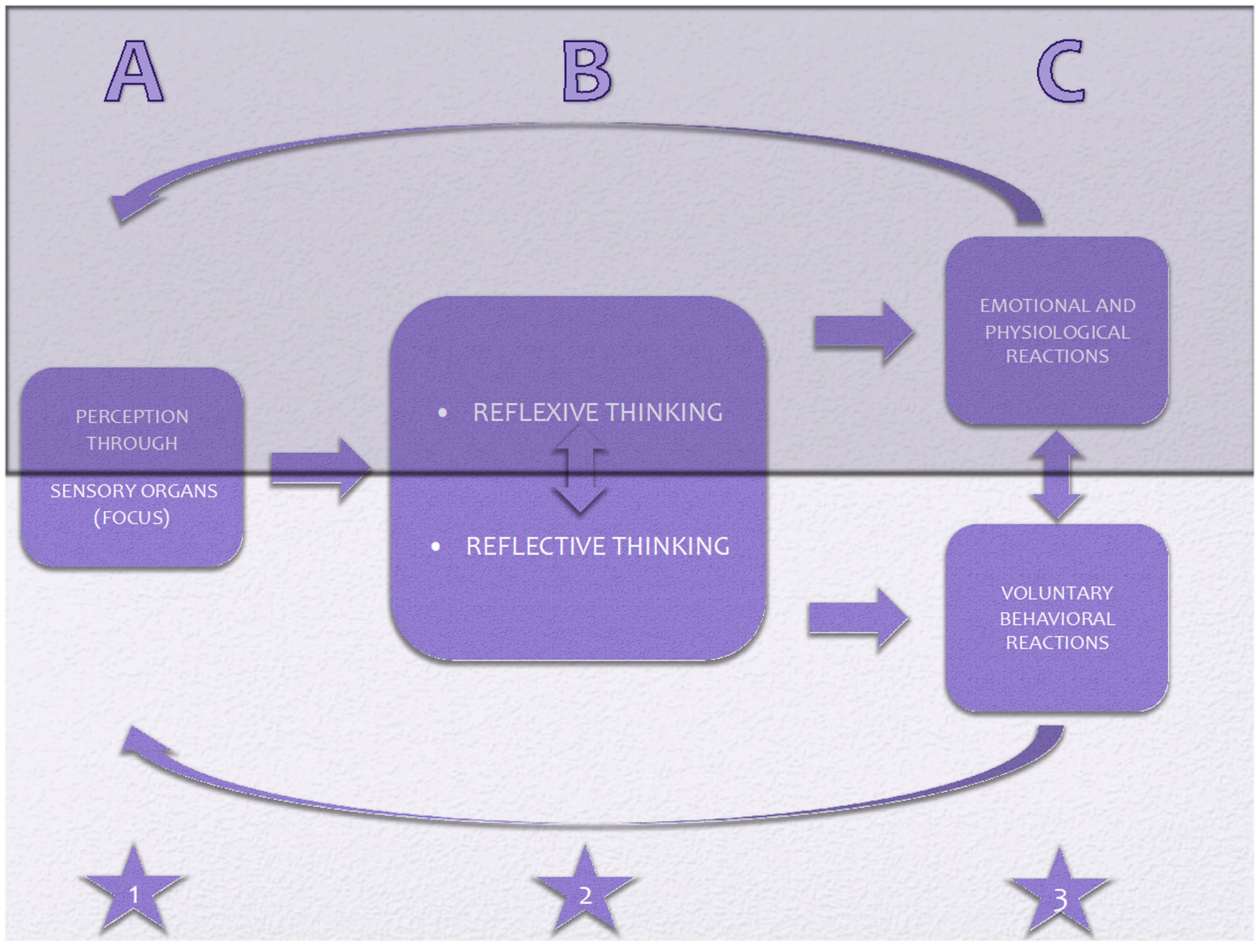
ABC of Tridimensional model: Three domains of reactions-including (attentional) focus domain (star 1), cognitive domain (star 2), and behavioral domain (star 3)-are portrayed in the figure. The darker part signifies involuntary/automatic components, whereas the lighter part remarks voluntary components. Oblique arrows above and below the figure represent the circular nature of the components since emotions, physiological, and behavioral reactions are perceived and processed all over.

Within the framework of this model, diagnosis-specific or transdiagnostic protocols can be developed, and interventions targeting these three areas can be implemented in modular formats. If these interrelated dimensions are key features in the maintenance of psychological disorders, the model must identify directly modifiable components to serve as an effective intervention framework. Some attentional and cognitive functions are automatic (e.g., attentional bias and intrusive thoughts), while others are intentional (e.g., threat monitoring and rumination) (Tolin, 2024). In the behavioral domain, emotional and physiological reactions are involuntary, whereas behaviors are voluntary. Table 1 provides examples of both involuntary and voluntary elements across the three domains. Figure 1 illustrates the Tridimensional model, showing the relationships between the three dimensions, their interconnections, and their automatic and strategic aspects.

**Table 1:** Examples of voluntary and involuntary reactions across the three domains.

Domain	Involuntary responses	Voluntary responses
Cognitive domain	Negative automatic thoughts	Worry and rumination (e.g., analyzing, fantasizing, self-criticism, searching for answers)
	Intrusive thoughts	Thought suppression, changing thoughts/images
	Intrusive images	Managing memories
	Memories	
Behavioral domain	Behavioral tendencies	Avoidance (e.g., isolation, passive behavior, sleeping)
	Behavioral inhibition	Safety-seeking behaviors, compulsions (rituals)
		Alcohol or substance use, overeating, Procrastination
Focus area	Automatic attentional shifts	Inwardly directed attention, threat monitoring

### How is it transdiagnostic and transtheoretical?

Although diagnostic developments that began in the late 19th century and reached their peak with the introduction of the DSM-III (APA, 1980) have provided a strong foundation for various clinical (e.g., diagnosis-specific protocols, randomized controlled trials) and scientific achievements (e.g., identifying neural circuits underlying obsessive compulsive disorder-OCD and anxiety disorders), the increasing number of psychological disorders listed in diagnostic manuals and the high rates of comorbidity among these disorders have led to questions about the validity of diagnosis-specific approaches. The transdiagnostic approach aims to identify and target underlying processes or mechanisms that are common across multiple mental disorders, rather than treating each diagnosis as a separate and distinct entity (Dalgleish et al., 2020; Roefs et al., 2022). In the literature, two main types of transdiagnostic approaches have been proposed: the “universal” approach, which applies the same mechanisms across a wide range of disorders, and the “modular” approach, which combines different intervention modules based on the individual’s specific symptom profile and needs (Schaeuffele et al., 2021). In this context, TriD-CBT is considered a universal model. In a study testing the assumptions of this transdiagnostic model, rumination, automatic thoughts, dysfunctional attitudes and thought suppression were found to be significant transdiagnostic factors (Yapan et al., 2022).

Previous efforts, such as Barlow’s Unified Protocol (Reinholt et al., 2017; Barlow et al., 2020), represent good examples of unified protocols. However, these protocols mainly rely on conventional behavioral and verbal techniques, rather than offering a truly transtheoretical approach, even within the CBT framework. There are other efforts to integrate or unify psychotherapy by combining elements from different approaches to enhance treatment effectiveness. Schema therapy (ST), for example, integrates elements from cognitive-behavioral, attachment, psychodynamic, and gestalt models. It utilizes techniques such as cognitive restructuring, experiential exercises (like the chair technique), limited reparenting, and behavioral pattern-breaking to help patients identify, understand, and modify maladaptive schemas and modes (Rafaeli et al., 2010; Brockman et al., 2023).

Dialectical behavior therapy (DBT) places emotion regulation and impulse control problems at the center of its model, integrating traditional CBT techniques with mindfulness interventions. DBT like ST is more than just a theoretical model; it is a comprehensive treatment package that includes skills training in mindfulness, distress tolerance, emotion regulation, and interpersonal effectiveness, as well as individual therapy, phone coaching, and therapist consultation teams, especially for people with borderline personality disorder (Lynch et al., 2006).

Another example is process-based CBT, which offers a framework for integrating different models that goes beyond traditional protocol-driven approaches. However, it is primarily a model for the individualization of the psychotherapy process. The main focus of this model is the acceptance and commitment therapy (ACT) approach. It also appears as a modular model for transdiagnostic integration (Hayes and Hofmann, 2021; Ryum and Kazantzis, 2024).

Labeling a model as “transtheoretical” can be somewhat problematic. In fact, our model does not address approaches outside of the cognitive-behavioral tradition, such as psychodynamic or attachment-based models like Emotion-Focused Therapy (Johnson, 2019). Rather, it aims to play a reconciliatory role among the various, and sometimes seemingly contradictory, schools within CBT. In line with the principle of parsimony (Epstein, 1984) the Tri-D model provides an economical, pragmatic, and practical framework for intervention and explanation by fundamentally integrating three domains—cognition, behavior, and attention—along with the distinction between voluntary and involuntary processes. Building on this theoretical and practical foundation, clinicians can draw upon both “traditional” cognitive-behavioral psychotherapies, such as Behavior Therapy (BT) (e.g., Wolpe, 1968), Rational-Emotive Behavior Therapy (REBT) (Ellis, 1984), Cognitive Therapy (CT) (Beck, 1967; Beck, 1979) and Cognitive Behavior Modification (CBM) (Meichenbaum, 1977), as well as “third-wave” cognitive-behavioral approaches, including Functional Analytic Psychotherapy (FAP) (Tsai et al., 2010), DBT (Linehan, 1993), Mindfulness-Based Cognitive Therapy (MBCT) (Teasdale et al., 1995), Behavioral Activation Therapy (BAT) (Dimidjian et al., 2011), ACT (Hayes et al., 1999), Compassion-Focused Therapy (CFT) (Gilbert and Irons, 2005; Gilbert, 2009), Metacognitive Therapy (MCT) (Wells, 2011), ST (Rafaeli et al., 2010) among others.

Our model is pragmatic, illustrating its main features through practical, concrete situations. In cognitive therapy, attention modification techniques are usually exceptions (Beck and Haigh, 2014)—such as situational attentional refocusing in the treatment of social phobia—whereas such techniques (Wells et al., 1997) are central interventions in MCT (e.g., Attention Training Technique). Balanced thinking and interventions aimed at achieving it (e.g., cognitive restructuring, generating alternative explanations) are at the core of every cognitive therapy protocol (Clark, 2013), whereas in ACT, such efforts are seen as signs of “fusion” and are to be avoided (Deacon et al., 2011). Behavioral approaches primarily focus on behavioral modification or re-learning through exposure, while cognitive and attentional domains are often addressed indirectly, such as by avoiding safety behaviors and ensuring clients are fully exposed for prolonged periods (Akkoyunlu and Türkçapar, 2013).

We operationalize our model as a universal framework in which all models and the techniques derived from them can find a place, allowing for their integration in a harmonious manner.

### How to apply the Tridimensional model for emotional disorders

In this model, the focus is on individuals’ reactions. Although biological and environmental factors are evident in all psychological disorders, the intensity, appropriateness, pervasiveness, and persistence of emotional reactions, as well as dysfunctionality in life areas, are the fundamental indicators of psychopathology (Kendall and Hammen, 1995). When using this model as an intervention framework, the emphasis is placed on the voluntary elements of the three dimensions. This distinction is particularly important because current diagnostic systems, such as the Diagnostic Statistical Manual of Mental Disorder 5th Edition (DSM-5) and International Statistical Classification of Diseases and Related Health Problems 11th Version (ICD-11), adopt a phenomenological approach (Regier et al., 2009). In these systems, both voluntary and involuntary reactions are often grouped together as symptoms of a disorder, without differentiating their functional roles. This can lead to a lack of clarity in understanding the mechanisms underlying the disorder and in designing targeted interventions. For instance, in anxiety disorders, an emotional response—such as fear—and a behavioral response—such as avoidance—are both included in the diagnostic criteria. However, while fear may be an involuntary reaction to a perceived threat, avoidance is often a voluntary coping strategy aimed at reducing distress.

The Tridimensional model challenges this traditional approach by emphasizing the need to separate voluntary and involuntary components, although both are considered symptoms of the disorder. This distinction is crucial because voluntary elements, such as avoidance or rumination, are often strategies employed by the individual to manage their distress, even if these strategies ultimately maintain or exacerbate the problem (Sahdra et al., 2016). In contrast, involuntary elements, such as physiological arousal or intrusive thoughts, are automatic responses that are not under the individual’s direct control.

For example, in OCD, compulsions are typically classified as symptoms of the disorder. However, from the perspective of the Tri-D CBT model, compulsions are better understood as voluntary coping strategies (as well as symptoms of the disorder) aimed at neutralizing the distress caused by obsessions or perceived threats. By distinguishing between these voluntary and involuntary components, the model provides a clearer framework for intervention. It allows therapists to target voluntary strategies for modification while addressing the involuntary reactions through other therapeutic techniques, such as exposure or cognitive restructuring.

This differentiation not only helps in creating a more nuanced understanding of the disorder but also aids in tailoring interventions to the specific needs of the individual. For instance, in the case of avoidance behaviors in anxiety disorders, the Tri-D CBT model would focus on reducing the voluntary avoidance strategies while simultaneously addressing the involuntary fear response through techniques like exposure therapy. Similarly, in depression, the model would distinguish between involuntary symptoms, such as low energy or intrusive negative thoughts, and voluntary strategies, such as rumination or withdrawal, which can be modified to promote recovery.

By separating voluntary and involuntary elements, the Tri-D CBT model offers a more precise and pragmatic approach to understanding and treating psychological disorders. This distinction not only aligns with the principles of cognitive-behavioral therapy but also enhances its effectiveness by ensuring that interventions are targeted at the most modifiable aspects of the disorder. However, strictly categorizing reactions as either “voluntary” or “involuntary” may not fully capture the complexity of strategies like rumination, which can involve both automatic and deliberate elements (Cann et al., 2011). Nevertheless, this limitation can be addressed through more nuanced explanations; for example, while the questions and triggering thoughts that prompt rumination are typically involuntary, the effort to find answers is more voluntary in nature.

**Figure 2:**
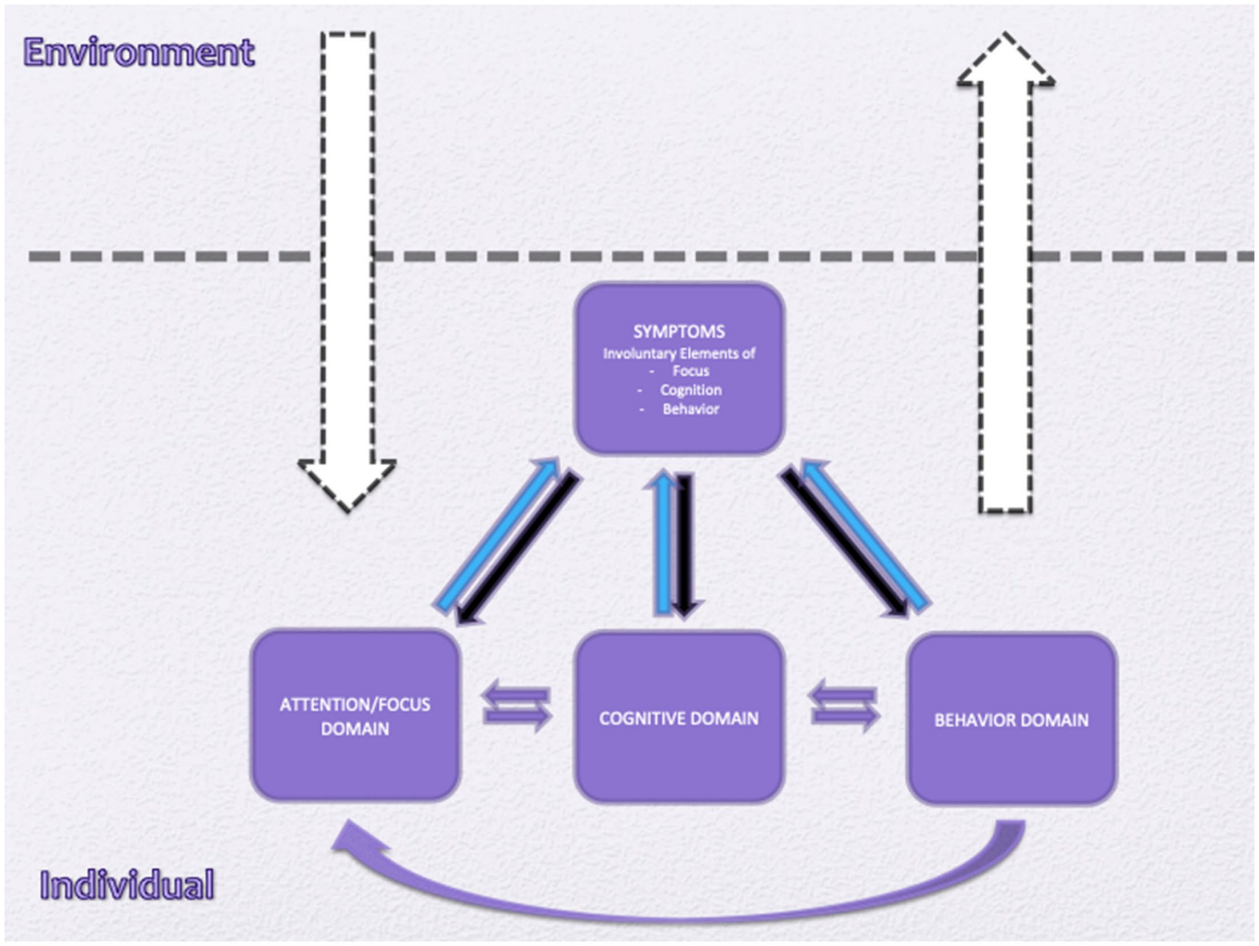
All individuals are influenced by their environment through the attention/focus domain and, in turn, affect their environment through the behavioral domain (white arrows). Individuals experiencing emotional disorders employ various strategies to cope with their problems, which are also considered symptoms of the emotional disorder. However, cognitive, attentional, and behavioral strategies are voluntary, even if the individual is sometimes unaware of them (black arrows). All strategies within the three domains act as maintaining factors for symptoms (blue arrows). Additionally, the three domains are interrelated (purple arrows).

The relationships within symptoms—including involuntary components and voluntary strategies—and their connections to the three domains of strategies are illustrated in Figure 2.

In TriD-CBT, the symptoms of a mental disorder that manifest as involuntary reactions of the organism are considered primary complaints. These include automatic thoughts, perceptions, and beliefs, as well as emotional and physiological responses. For example, body image disturbance in anorexia nervosa (AN) and worthlessness beliefs in depression are considered primary complaints. In depression, symptoms such as loss of interest, insomnia, low energy, negative memories, thoughts of regret, and the belief that one is unloved are also included. However, behaviors like social isolation or avoidance are not considered pure symptoms, as they are both symptoms and coping behaviors. These are categorized under the behavioral dimension of the tridimensional formulation.

As mentioned earlier, the model consists of three response areas: cognitive, attentional, and behavioral domains. These three domains represent the individual’s reactions to alleviate existing symptoms. However, these reactions often backfire, either in the short term (e.g., rumination) or long term (e.g., avoidance).

#### Cognitive domain

Both negative automatic thoughts and repetitive thinking (e.g., worry and rumination) are associated with and predictive of negative affect (McLaughlin et al., 2007; Fatih et al., 2020). Cognitive restructuring, one of the fundamental techniques of cognitive therapy, involves recognizing automatic thoughts that lead to or accompany negative emotions and developing more realistic, appropriate, and functional thoughts to replace them. This process enables individuals to consider data that may have been overlooked due to emotional intensity, thereby allowing for more effective cognitive processing. In other words, it aims to reappraise the event or situation.

From another perspective, this process is the opposite of ruminating or worrying (repetitive thinking). In worry and rumination, the individual engages in a dysfunctional thought process using biased data and incomplete input. Models based on behavioral and metacognitive theories focus on controlling rumination and worry processes. For example, distraction and mindfulness-based interventions attempt to prevent triggering thoughts from progressing into a ruminative process. The goal is not to eliminate the negative emotion associated with a momentary thought or to quickly change the thought itself. Efforts to suppress or quickly change negative emotions or thoughts are considered dysfunctional in most models. This is particularly evident in interventions for OCD and anxious thoughts.

One of the three obsessive belief domains defined by the OCD Study (Group O. C. C. W, 1997) involves the importance and control of thoughts. Interventions under the CBT umbrella aim to either purify the thought process from cognitive biases or prevent dysfunctional thinking processes (e.g., worry, rumination, self-criticism) more directly (Aldao et al., 2010; Brozovich et al., 2015). Another focus of intervention in this domain is limiting or banning mental operations that aim to suppress or neutralize negative thoughts or images. Mental operations like gap-filling are also included in this domain (Wells, 2011).

Some research suggests that reducing ruminative thinking is more strongly associated with symptom reduction than reappraisal techniques (Nolen-Hoeksema and Watkins, 2011). Several studies indicate that rumination and worry are more closely related to depression and anxiety than reappraisal (Brozovich et al., 2015). Additionally, findings suggest that reappraisal and rumination can transform into one another (Brozovich et al., 2015).

In summary, functional reactions in this domain include reappraisal (not prolonged as a repetitive thinking process), balanced thinking, accepting thoughts, and limiting repetitive thinking. Dysfunctional reactions, such as repetitive/prolonged thinking (worry and rumination), thought suppression (Wegner et al., 1987), cognitive avoidance, neutralization, and gap-filling, are promoted to decrease (Folkman and Lazarus, 1986; Clark, 2006; Williams and Moulds, 2007; Wells, 2011; Brozovich et al., 2015).

#### Behavioral domain

No behavior can be labeled as “dysfunctional” or “pathological” without considering its context and outcome. When examining behaviors, it is essential to evaluate the context of prior intentions, expectations, and subsequent results. From a behavioral perspective, a behavior increases or decreases based on reinforcement through its consequences. New behaviorism and social learning theories contribute to this perspective by arguing that expectations before the behavior can modulate the effect of consequences on the behavior. Social learning is a cognitive process that increases the likelihood of a behavior occurring (Bandura, 1999). Expectations and self-efficacy influence how reinforcing the consequences of a behavior will be.

Behaviors in mental disorders are considered dysfunctional due to their direct consequences (e.g., compulsions consuming time or avoidance narrowing the person’s living space) and the negative reinforcement of the behavior through continued learning processes (e.g., reducing negative emotions through compulsion or avoidance). Exposure treatments aim to reduce dysfunctional behaviors (e.g., avoidance, safety-seeking, or compulsive behaviors) and increase functional behaviors (e.g., problem-solving, engaging in personally meaningful activities). Paradigms such as reconditioning, extinction of conditioning, or inhibitory learning have been proposed to explain the mechanisms underlying the positive effects of these treatments (Craske et al., 2014).

Cognitive and behavioral domains mutually influence and change each other. From a cognitive perspective, the main consequence of dysfunctional behavior is the failure to test underlying beliefs that contribute to the problem. For example, an individual who believes they will remain distressed all day if they do not wash their hands or who fears getting sick without performing a ritual is deprived of testing these beliefs by engaging in compulsions and safety-seeking behaviors (McMillan and Lee, 2010). Conversely, testing functional behaviors can lead to cognitive change by challenging underlying beliefs (Foa and Rauch, 2004). For instance, when a person who believes they cannot lose weight even with a diet turns dieting into an experiment, they test their belief that “If I follow the diet, I will be rewarded.

In summary, active problem-solving and behavioral strategies consistent with actual threats are functional and should be promoted, while avoidance, safety-seeking behaviors, and compulsive rituals are dysfunctional and should be reduced.

#### Focus (attentional) domain

Although the focus domain was emphasized in the early stages of schema theory, it did not gain much prominence as an intervention area in the early years of CT. Since schemas are defined as cognitive structures that influence an individual’s attention, perception, evaluation, and predictions, it is known that when a certain schema is activated, the individual exhibits an attentional bias favoring data consistent with the schema (Lawson et al., 2002). However, starting in the 1990s, focus and attention began to gain significant attention in psychotherapy, particularly with the advent of mindfulness-based practices and MCT. Recent developments have also been supported by neuroimaging studies, which consistently demonstrate the central role of the amygdala in modulating attentional biases associated with anxiety and depression. Research indicates that heightened amygdala activity is linked to increased vigilance toward threat-related stimuli in anxiety, while altered amygdala-prefrontal connectivity has been implicated in the persistent negative attentional focus observed in depressive disorders (Etkin and Wager, 2007; Disner et al., 2011). These findings underscore the bidirectional relationship between attentional bias and emotional symptoms, suggesting that interventions targeting amygdala-related neural circuits may be effective in alleviating both anxious and depressive symptomatology (Siegle, 1999; Van Bockstaele et al., 2014). In these years, techniques aimed at directing attention also emerged as independent healing factors for psychopathologies (Eysenck et al., 2007; Amir et al., 2009).

In their seminal article, Beck and Haigh (2014) proposed focus as an additional element alongside beliefs and behaviors. However, they did not assign it a specific role within the theory. Attentional issues in psychopathologies can manifest in various forms, including self-focused attention, threat monitoring, or distraction. Self-focused attention is a particularly significant construct, defined as an awareness of internal stimuli rather than external stimuli gathered through sensory organs. Internally, these stimuli consist of self-referent information. Whether disordered or not, when someone experiences distress, attentional focus tends to shift toward the inner emotional environment (Ingram, 1990).

Woodruff-Borden et al. (2001) and Harvey et al. (2004) revealed that self-focused attention is a critical element in many psychological disorders, including depression and various anxiety disorders. Woodruff-Borden et al., found that negative self-focus was strongly related to the severity of psychopathology in individuals with various psychological disorders, including clinical anxiety and depression. Another important finding of their study is that self-focused attention is negatively correlated with problem-solving abilities (Woodruff-Borden et al., 2001).

There is a bidirectional relationship between self-focused attention and negative affect (Ingram, 1990; Brune and Brune-Cohrs, 2006; Hinds et al., 2012). Woodruff-Borden et al., suggested that this process could lead to a ruminative cycle (Woodruff-Borden et al., 2001). Similar to other findings, the inflexibility of attention, its intensity, and self-focused attention—rather than the cognitive content itself—appear to predict the pathological nature of cognitions.

Many other studies have suggested the etiological role of threat monitoring (Shechner and Bar-Haim, 2016). In depression, individuals are prone to focus on and engage with negative, dysphoric external stimuli, while they do not spontaneously concentrate on threatening stimuli. However, in anxiety disorders, individuals are prone to spontaneously focus on threatening stimuli and have difficulty disengaging from them (Armstrong and Olatunji, 2012). Other studies have shown that threat monitoring, whether the threats are internal or external, is a maintaining factor for emotional disorders (Bardeen et al., 2022).

In this context, flexible attentional control and balanced attention (between safety and threat) are potential therapeutic goals, particularly when self-focused attention and threat monitoring are dysfunctional strategies that need to be diminished.

#### What are the differences from previous approaches?

The first and most important point is that each model tends to emphasize its own distinct features, often overlooking the effective practices of others. For example, ACT avoids working on the content of beliefs (Hayes et al., 2006) while MCT almost entirely rejects reappraisal, viewing it as a form of repetitive thinking. Similarly, CT does not place central importance on attentional functions (Fisher and Wells, 2009).

CBT is distinguished by its integrative approach, which contributes to its effectiveness (Alford and Beck, 1998). Hayes and Hofmann (2021) emphasize that a progressive field incorporates what is helpful from previous approaches and carries it forward, allowing these approaches to evolve in a broader and more interconnected way. Tolin (2024), on the other hand, argues that intervening in one of the interlinked domains may have a snowball effect, influencing other domains as well. Taken together, these considerations highlight the potential drawbacks of excluding certain theoretical and clinical approaches or practices in the pursuit of so-called brand-new therapies.

The main contribution of our approach is the simplification of integration across different models, particularly since many models either do not provide space for certain techniques (such as reappraisal in MCT) or actively avoid them (such as attention modification in cognitive therapy). Additionally, our model uniquely and explicitly differentiates between voluntary and involuntary strategies across three domains.

## Discussion

In summary, the main hypotheses of this model are:

a) From a cognitive-behavioral perspective, the difficulties associated with any psychological disorder can pragmatically be categorized into three domains.
b) Every domain has voluntary and involuntary components.
c) The three domains of operations are interrelated with each other.
d) The TRIDIMENSIONAL model suggests a framework to integrate various models and interventions originating from the theories under the CBT umbrella.

Symptoms of psychological disorders (mainly involuntary ones) prompt individuals to react through three main domains of operation: attentional, cognitive, and behavioral. Dysfunctional tendencies such as threat monitoring, repetitive negative thinking, biased evaluation, and avoidance are evident across all these domains. Moreover, dysfunctional operations in these three domains exacerbate the symptoms of psychological disorders. This bidirectional relationship highlights the key areas that are candidates for change in all cognitive-behavioral interventions.

Since one of the functions of repetitive thinking is to find ways to avoid feared stimuli (Borkovec and Roemer, 1995), both cognitive and behavioral operations are inherently interrelated. Research on the dyadic relationships between the three domains suggests that cognitive, behavioral, and attentional (focus) domains are interconnected (Wisco and Nolen-Hoeksema, 2008). This relationship is also emphasized in Wells’ metacognitive model, where these three domains are collectively referred to as the “Cognitive Attentional Syndrome (CAS)” (Wells and Matthews, 1996). In his model, Wells proposed that all CASes stem from specific metacognitions regarding control and perceived danger.

All learned experiences and their semantic associations are reflected in the “representation system,” which operates beyond the generic memory definition (Chein and Schneider, 2012). This system functions slowly but in a more stable manner. In contrast, the “cognitive control network” guides attention and cognitive processing, enabling goal-directed, sequenced behavioral tasks. The “metacognitive system” oversees the cognitive control network. However, in the Tri-Dal model, cognitive, behavioral, and attentional operations are central and are not necessarily tied to metacognitions. Avoiding the debate on whether cognitive or metacognitive change is at the core of learning, the model instead emphasizes these three domains (cognitive, behavioral, and attentional) as shared by the main theories. All interventions are expected to target one, two, or all three of these domains. More specifically, all strategies—regardless of their origin—should aim to modify voluntary components within the focus, cognition, and behavior domains.

The process of controlled cognition is closely associated with attentional mechanisms (Chein and Schneider, 2012). Over time, repeated exposure to stimulus–response pairs can lead to the development of automatic attentional patterns and processing, which demand minimal cognitive effort. This is particularly relevant for clinical conditions where attentional, cognitive, and behavioral strategies become increasingly automatic over time. In such cases, awareness of the trigger stimuli often increases, while awareness of dysfunctional strategies decreases. For example, avoidance strategies are closely tied to automatic attentional biases toward avoided stimuli (Luecken et al., 2004). Emotional load often causes attentional bias, leading attention to withdraw toward perceived threats. If an individual does not employ a functional strategy, they cannot access safety information (Beck and Clark, 1997; Lohr et al., 2007).

Considering the discussion above, the three main domains of reaction—cognitive, behavioral, and attentional—play a key role in maintaining psychopathology. Thus, all interventions or techniques need to address these domains. Successfully delivering techniques in CBT practice requires synergy across these three domains. For instance, having a client engage in cognitive restructuring could easily turn into a process of worry or rumination unless balanced attentional strategies are employed. Similarly, it could function as an avoidance strategy if the individual continues to engage in safety-seeking behaviors (Krafft et al., 2022). These interrelationships are evident in both depression and various anxiety disorders (Sloan and Telch, 2002; Newby and Moulds, 2010).

Another example is behavioral activation (based on behavioral model of depression) interventions in depression therapy. Effective behavioral activation practices should involve an external attentional focus and discourage ruminative thinking styles (Schmitter et al., 2023). The interrelated nature of the three domains has been demonstrated in studies conducted on both normal populations (Dickson et al., 2012) and clinical populations with depression and anxiety disorders (Naragon-Gainey et al., 2017). However, the extent to which these strategic domains and related strategies are deployed varies across psychopathologies (Aldao et al., 2010).

In a meta-analytic review, Aldao et al. (2010) found that the effect sizes for rumination were large, while the effect sizes for avoidance, problem-solving, and suppression were medium to large. In contrast, the effect sizes for reappraisal and acceptance were small to medium. These findings suggest that while all three domains are critical, the relative importance of each domain and its associated strategies may differ depending on the specific psychopathology.

## Conclusion

The T TriD-CBT model offers a practical and pragmatic framework for integrating interventions and techniques under the umbrella of CBT. It emphasizes that focusing, thinking, and behavioral reactions are the core elements for understanding psychological problems and organizing treatment interventions to address them. In contrast to rigid school orthodoxy, TriD-CBT suggests that all approaches can be understood and implemented within this pragmatic model. The model’s aim is not to blend all existing models into one, but to offer a foundation for integration based on their essential commonalities.

We hope that TriD-CBT can assist therapists in reconciling seemingly contradictory models by providing a unifying perspective. For example, consider a client with social anxiety who describes himself as “a loser” during a therapy session and recounts a situation where he felt extremely anxious and feared humiliation. In that situation, his attention was self-focused; he initially tried to appear “calm” but eventually left the situation. When working with this client, a therapist might adopt a purely behavioral approach, using exposure practices as foundational tools; a radical behaviorist approach, such as ACT, employing defusion techniques and value-based behaviors as primary tools; or a cognitive approach, utilizing cognitive restructuring and behavioral experiments as prominent tools. Let us assume that the client also has major depressive disorder (MDD) and holds the belief that nothing is going to change. A behaviorist would conceptualize the hopelessness as an internal event which is not directly touchable and instead focus on observable behaviors such as social isolation, procrastination and passivity. The intervention mainly would be behavioral activation, encouraging the client to gradually increase engagement in meaningful or pleasurable activities, with the aim of breaking the cycle of inactivity and low mood.

A cognitive therapist would address the client’s negative belief directly, using techniques such as cognitive restructuring to challenge and modify the thought that “nothing is going to change.” Alternatively, he/she designs behavioral experiments, encouraging the client to test out that belief in real-life situations—for example, by engaging in a new activity and observing whether it leads to any change, thereby gathering evidence that contradicts the original belief.

A radical behaviorist, drawing from ACT, would focus more on altering their relationship to those thoughts. They might introduce cognitive de-fusion techniques to help the client see thoughts as mental events rather than absolute truths, and encourage actions guided by personal values, even in the presence of difficult emotions or beliefs.

A MCT therapist would take a different approach, targeting the processes that maintain depressive thinking and metacognitions behind them. They might implement the Attention Training Technique (ATT) to help the client test out “uncontrollability metacognitions” and gain flexible control over their focus of attention, and introduce strategies such as postponing rumination, teaching the client to delay or limit the time spent dwelling on negative thoughts.

If the client also struggles with binge eating disorder, each model would adapt its interventions accordingly. A behaviorist might focus on identifying and modifying environmental triggers for binge eating and reinforcing alternative, healthier behaviors. A cognitive therapist would explore and challenge dysfunctional beliefs about food, body image, and self-worth, possibly using thought records and behavioral experiments related to eating patterns. An ACT therapist would work on increasing acceptance of cravings and emotions, while helping the client commit to value-driven eating behaviors. A MCT therapist might address unhelpful metacognitive beliefs about monitoring their body, repetitively thinking about calories and implement techniques to limit worry and rumination.

In this context, third-wave cognitive-behavioral psychotherapies represent an extension of the theoretical foundations of the CBT tradition, incorporating new and diverse scientific approaches. This development enables researchers and clinicians to adopt an integrative approach that remains theoretically consistent, which is a significant advantage. However, those who expand upon this foundation with distinct approaches or perspectives may (I) over-differentiate and overemphasize their own approach or so-called “brand” of therapy, or (II) ignore or neglect other important elements of the theoretical background in an effort to distinguish their particular “therapy” brand. These tendencies can be seen as disadvantages.

In summary, while each therapeutic model brings its own perspective and techniques, an integrative or transdiagnostic approach can help tailor interventions to the client’s unique needs, drawing on the strengths of each tradition. From the TriD-CBT perspective, the client must learn to shift attention away from internal and threatening external stimuli and remain in the avoided situation for a longer period and take actions that beneficial for him in a long run. Over time, this allows the client to gather additional balanced information, reflect on new insights, and reappraise the situation. Alternatively, the client may stop worrying/ruminating (about socially anxious situation, hopelessness, gaining weight), allowing the automatic evaluation process to take over.

As a limitation, while the model appears practical and straightforward, it is grounded in highly complex scientific fields such as cognitive psychology, learning theory, and psychopathology. Therefore, we recommend that the assumptions presented here be tested in future studies. We believe that examining the proposed model through theoretical testing and developing practical guidelines for its clinical application will significantly contribute to the field of psychotherapy.

### Future directions

In essence, the TriD-CBT model offers a comprehensive framework for applying CBT practices and techniques, rather than prescribing which specific interventions should be used—at least for now. The next step, however, would be to develop concrete and robust techniques derived from the TriD-CBT approach. For example, the rumination or worry postponement technique can be integrated with cognitive reappraisal strategies. In this approach, the client first identifies their negative automatic thought during moments of heightened emotional arousal. Since attention is likely to be biased at this time, the primary task is to redirect attention externally, preferably toward neutral or non-threatening stimuli. The client is then encouraged to postpone engaging with the trigger thought and its associated emotional or physiological reactions, as immediate cognitive processing may easily lead to repetitive negative thinking. Once the emotional intensity has naturally subsided—typically after half an hour to two hours—the client can then analyze the thought in terms of its rationality, compatibility, and function.

Within the TriD-CBT framework, these modified techniques can be empirically compared to classical cognitive restructuring—which is usually implemented immediately after the triggering event—as well as to interventions from BT, MCT, and ACT in experimental studies. This approach allows for a systematic evaluation of the effectiveness and applicability of integrated techniques across different therapeutic traditions. In this regard, every well-established technique can be adapted and examined through a three-dimensional focus, allowing for systematic testing and refinement within this integrative framework.

Case studies can further illustrate how case formulations and treatment plans based on the TriD model are implemented in clinical practice, by focusing on the voluntary and involuntary components of the cognition, attention, and behavior domains proposed by the model. The efficacy and effectiveness of treatment manuals or techniques modified or customized according to the TriD framework can be tested through clinical trials, such as randomized controlled trials. More specifically, it can be investigated whether utilizing a holistic and pragmatic TriD paradigm—rather than relying solely on one of the competing variations of CBT—during different phases of therapy, such as evaluation and psychoeducation, leads to improved treatment outcomes.

Additionally, we recommend developing and testing models that are not constrained by the theoretical assumptions of diverse psychotherapy approaches, but are instead extended with the TriD framework. In this way, we highlight the potential for creating theories and practices that are more grounded in real life and possess higher external validity.

## Data Availability

The original contributions presented in the study are included in the article/supplementary material, further inquiries can be directed to the corresponding author.
